# Energetic Lifestyle Drives Size and Shape of Avian Erythrocytes

**DOI:** 10.1093/icb/icab195

**Published:** 2021-09-28

**Authors:** Carl D Soulsbury, Jessica Dobson, D Charles Deeming, Piotr Minias

**Affiliations:** School of Life Sciences, University of Lincoln, Brayford Pool, Brayford Pool, Lincoln LN6 7TS, UK; School of Life Sciences, University of Lincoln, Brayford Pool, Brayford Pool, Lincoln LN6 7TS, UK; School of Life Sciences, University of Lincoln, Brayford Pool, Brayford Pool, Lincoln LN6 7TS, UK; Department of Biodiversity Studies and Bioeducation, Faculty of Biology and Environmental Protection, University of Łódź, ul. Uniwersytecka 3, 90-137 Łódź, Poland

## Abstract

The size and shape of red blood cells (erythrocytes) is determined by key life history strategies in vertebrates. They have a fundamental role to deliver oxygen to tissues, and their ability to do so is shaped by the tissue's need and their shape. Despite considerable interest in how other components of blood are shaped by ecology and life history, few studies have considered erythrocytes themselves. We tested how erythrocyte size and shape varied in relation to energetically demanding activities using a dataset of 631 bird species. We found that in general, birds undergoing greater activities such as long distance migration had smaller and more elongated cells, while those with greater male-male competition had smaller and rounder cells. Smaller, more elongated erythrocytes allow more rapid oxygenation/deoxygenation and support greater aerobic activity. The rounder erythrocytes found in species with strong male–male competition may stem from younger erythrocytes deriving from androgen-induced erythropoiesis rates. Finally, diving species of bird had larger erythrocytes, indicating that erythrocytes are acting as a vital oxygen store. In summary, erythrocyte size and shape in birds are driven by the need to deliver oxygen during energetically costly activities.

## Introduction

Blood oxygen-carrying capacity is one of the major determinants of the amount of oxygen delivered to tissue per unit of time, and therefore has a critical role in shaping oxidative metabolism (Minias 2020). Blood oxygen-carrying capacity is often measured as total hemoglobin (Hb) concentration in blood and hamatocrit (Hct). Hb measures the total amount of hemoglobin in the blood, whereas Hct measures the percentage of red blood cells in a volume of blood. Experimentally, within species studies have shown that Hct and Hb were significantly lower after long-term exercise training (Bury et al. 2019), and Hct was lower after higher altitude acclimatization (Yap et al. 2018). Similarly, across species there is strong evidence that both Hct and Hb are adapted to a range of differing life history strategies, such a migratory strategy, altitude, and metabolic rate (Minias et al. 2013; Yap et al. 2019; Minias, 2020). In combination, these examples highlight the critical role behaviors and life history have in shaping blood oxygen-carrying capacity. While Hb and Hct are by far the most widely studied measures of blood oxygen-carrying capacity, a number of other components of blood oxygen-carrying capacity, such as erythrocyte size and shape, are important and major targets of selection.

Across all vertebrates, there is considerable variation in erythrocyte size between taxa (Hawkey et al. 1991; Lay and Baldwin 1999; Gregory 2001). A major driver of these differences comes from the positive relationship between genome size and cell size (Gregory 2000, 2001, 2002), which is directly related to nucleus size in vertebrates with nucleated erythrocytes (Gregory 2002). Genome size is linked to metabolic rate (Gardner et al. 2020), and across taxa, ectothermic vertebrates (fish, reptiles, and amphibians) have larger cells than endothermic mammals and birds (Glomski and Pica 2006). Similarly, within-taxa differences in erythrocyte size scale with body mass in amphibians and birds (Kozłowski et al. 2010) and squamates (Penman et al., submitted for publication). In many species, there are ontogenetic changes in erythrocyte size (reptile: Starostová et al. 2013; fish: Lahnsteiner 2020) that may stem from changes in mass-specific metabolic rate (Pis 2008).

A second major driver of differences in erythrocyte size stems from components of organism's ecology and life history. For example, individuals living at different altitudes have very differing erythrocyte sizes both within species (mammals: Tufts et al. 2013; squamates: Gonzáles-Morales et al. 2015) and more broadly across taxa (anurans: Navas and Chauí-Berlinck 2007; squamates: Penman et al., submitted for publication). Moreover, erythrocyte size can show plastic changes related to seasonal movements up and down altitudinal gradients (Janiga et al. 2017), temperature (Ruiz et al. 2004), responses to environmental contaminants (Janiga and Haas 2019), and to between- and within-year changes in energetic-demands, such as moulting (Haas and Janiga 2020) or mating effort (Buckneret al., submitted for publication). Other sources of variation in erythrocyte size are less well studied. For example, in teleosts, species with higher aerobic capacity and greater activity levels have smaller erythrocytes (Wells and Baldwin 1990), and active foraging squamates have smaller erythrocytes (Penman et al., submitted for publication). More broadly across teleosts, erythrocyte shape was unrelated to habitat or body size (Martins et al. 2021). In summary, existing work has looked at erythrocyte size and shape both broadly and in a handful of specific studies, for specific drivers.

To date, several studies have considered how blood oxygen-carrying capacity, specifically Hct and Hb, are related to components of an organism's life history and energetic lifestyle in birds (Yap et al. 2019; Minias 2020), but as yet no studies have considered how energetic lifestyle may influence erythrocyte size and shape. Birds have a very different respiratory systems to other air-breathing vertebrates with highly vascularized but non-distendable lungs and nine avascular air sacs not involved in gas exchange (Maina 2005). The air sacs are membranous structures that, in some species, extend between the muscles and even enter the bones (Butler et al.^1988^). Inspired air moves via the parabronchi to the posterior air sacs and bypasses the lungs entirely. Expiration sees the air move into and through the lungs, with the unidirectional flow supported by aerodynamic valves. Upon the next inspiration, the air moves into the anterior air sacs and eventually leaves the body at the next expiration (Maina 2002; Butler et al. 1988). By contrast, most other vertebrates have tidal flow respiration. The mammalian conducting airways arborize with the branch tips ending in blind sacs, there are no valves, and gases exhibit tidal flow by travelling in the opposite direction along the conducting airways during expiration from the direction followed during inspiration (Hill et al. 2016).

The presence of aerodynamic valves and unidirectional air flow has generally been thought to be a highly derived feature found, among extant animals, only in birds and crocodilians (Farmer and Sanders 2010; though see Cieri et al. 2014). This system has thought to have evolved as a mechanism to meet the high aerobics demands of endothermy and specifically flight (Farmer 2006). In addition, other components of cardiovascular system are also adapted to aerobic capacity, e.g., heart size is related to flight style (Nespolo et al. 2018). Hence, it seems plausible that components of the blood oxygen-carrying capacity may also vary where aerobic demand is higher. To date, studies using Hct and Hb have indeed found that species with aerobically demanding behaviors, e.g., diving, show concomitant changes in blood values (Yap et al. 2019; Minias 2020). However, it is not known if this also affects erythrocyte size.

In this study, we used data for erythrocyte area and elongation ratio (i.e., length/breadth) from 631 species of birds and tested whether a number of key ecological (migration and altitude) and behavioral (diving behavior and mating system) factors that are all intrinsically intertwined with energetic lifestyle have shaped erythrocyte size and shape.

## Methods

### Data collection

We gathered data for erythrocyte area (µm^2^), length along the long axis (μm), and breadth of the short axis (μm) from numerous sources in the published literature. Where area was not reported, we calculated the area based on length × breadth measurements of an ellipsoid. We focussed on erythrocyte area, rather than mean corpuscular volume, which is usually derived by multiplying a volume of blood by the proportion of blood that is cellular (the hematocrit), and dividing that product by the number of erythrocytes (red blood cells) in that volume. This misses key information on cellular shape, which has a functional role in blood flow. We calculated an elongation ratio (i.e., length/breadth) for erythrocytes so as to assess how the shape of erythrocytes changed. Erythrocyte size data were collected from the literature, and as a consequence there will be study-specific differences in handling, and processing that can impact erythrocyte size (e.g., shrinkage). Between-study methodological difference will contribute to unquantified variance in our data, and further work should assess how great this effect might be.

### Ecological data

Too few studies gave specific locations of capture, as many were collected for parasitological studies and reported uninfected erythrocyte size. We included key ecological variables including total migration distance (1 + log_10_ transformed in models) and breeding latitude [see Minias (2020) for methodology]. Without specific sampling locations, we could not include specific altitudes of specimen collection. Instead, we included the maximum recorded elevation for focal species taken from (Quintero and Jetz 2018).

### Mating system data

We also included information on bird mating systems from Dunn et al. (2015), supplemented where possible from information included in Birds of the World (https://birdsoftheworld.org/bow/home). Social and genetic mating systems may differ substantially, so we included relative testes size as a measure of male–male competition, based on body mass and testes size measures (Dunn et al. 2015). Methods for testis mass data collection are outlined in Dunn et al. (2001); data were collected from at least 5 males from the breeding season (Dunn et al. 2001). Body mass was used as the mean value for the species.

### Data processing and statistical analysis

Our initial dataset contained a clear set of outliers of species measured from papers by Savage and Greiner (2004) and Savage et al. (2005). It is not clear why data from these two papers were outliers, but as they both came from same authors, it is possible there is some systematic error within these data so we excluded these values (N = 4 species). Few samples came with specific information on location, sex of sample or data on body size. To reduce residual variation driven by lack of variance in predictor variables, we used single values for each species, and where sex was known, values for males were included because of the analyses focusing on relative testes size and mating system.

We created an initial dataset of 631 bird species. It was not possible to run a single analysis, due to missing data among predictor variables, so we carried out two sets of different models covering differing available information. For each model, we tested how erythrocyte size (area, log_10_ transformed) and elongation ratio (length/breadth) was related to set of ecologicals or life history variables. In the first pair of models, we tested whether erythrocyte area and elongation ratio were related to body mass (erythrocyte area only, as elongation ratio does not scale with body size), altitude (maximum recorded altitude in m), diving behavior (yes/no), and either migration distance (km, 1 + log_10_ transformed) or latitude (absolute degrees from equator). Predictor variables showed no collinearity, though latitude and migration distance were moderately correlated (*r* = 0.58, *P* < 0.001), so each variable was run in a separate model. The second pair of models examined the effects of relative testes size (i.e., testes mass/body mass) and mating system, i.e., cooperative, lekking, monogamous, polyandrous, polygynous, or promiscuous breeding patterns, on erythrocyte area and elongation ratio. With the exception of body mass, the same covariates were investigated for elongation ratio.

To account for phylogenetic relatedness within our dataset, we downloaded 100 trees from birdtree.org (Jetz et al. 2012) and summarized the phylogenetic information into a 50% majority‐rule consensus tree. We carried a phylogenetic generalized least square regression (Freckleton et al. 2002). We estimated Pagel's λ within the model, where λ = 0 represents no evolutionary signal (no covariance in the residuals due to shared ancestry), and λ = 1 indicates that the observed covariance in residuals follows that expected under a Brownian motion model of trait evolution. All models were run in R version 4.0.3 (R Core Team 2020).

## Results

### Geographical and aerobic activities

**Fig. 1:**
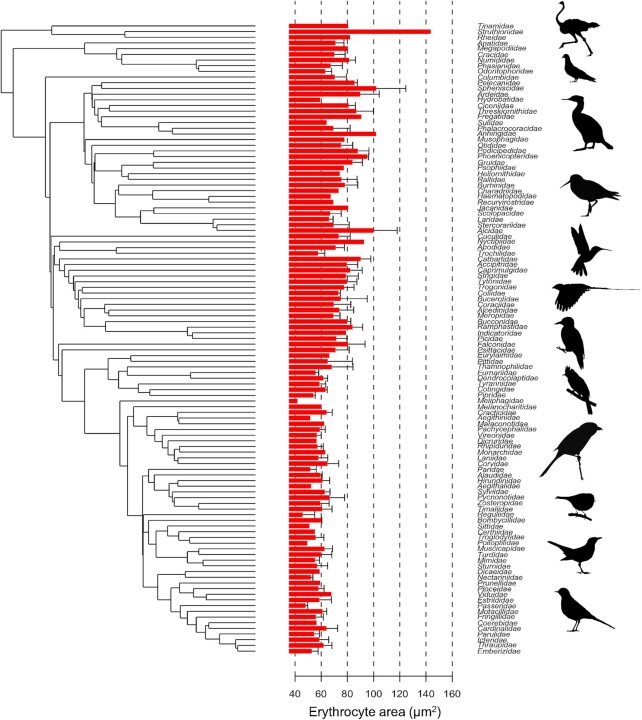
Mean (+ standard deviation) values for erythrocyte area of different bird families. Phylogenetic relationships for the different families are shown according to Jetz et al. 2012. Images are taken from Phylopic (http://phylopic.org) with full links and detail in the acknowledgments.

Our data had a broad taxonomic coverage, with 629 species, coming from 111 of the 249 bird families (44.6%) and 31/36 avian orders. Erythrocyte size varied considerably, being smallest in the golden-crowned kinglet *Regulus satrapa* (38.59 µm^2^) and largest in the ostrich *Struthio camelus* (143.70 µm^2^; Fig. 1).

**Fig. 2:**
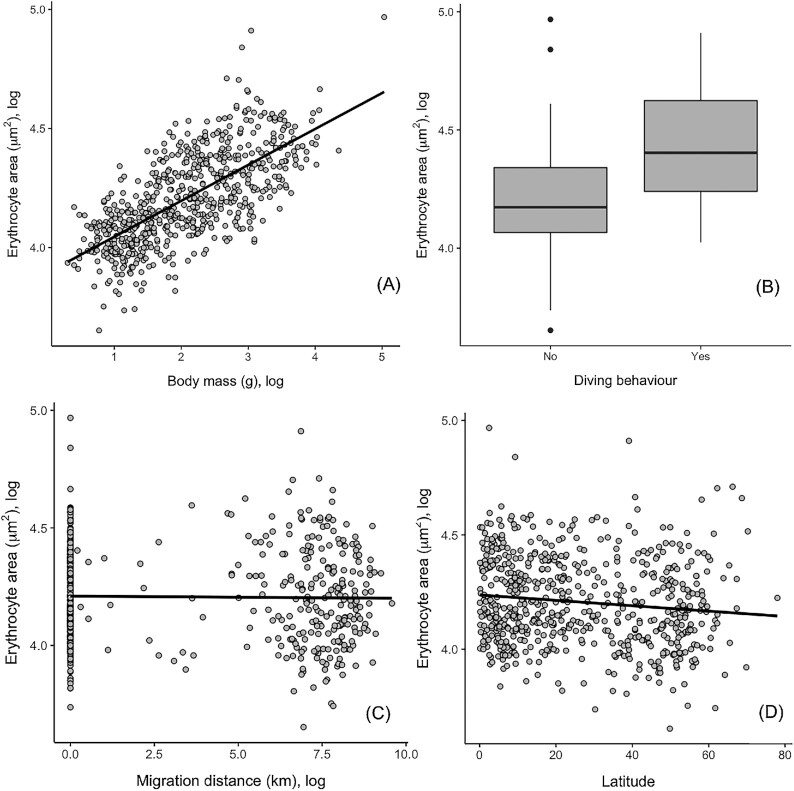
Effects of (**A**) body mass (log g), (**B**) diving behavior, (**C**) total migration distance (log km), and (**D**) absolute latitude (degrees from equator) on erythrocyte area (μm²).

Heavier and diving species had significantly larger erythrocytes (Table 1; Fig. 2A). Erythrocyte size was not related to maximum reported altitude, but there was a significant negative effect of migration distance on erythrocyte size (Table 1; Fig. 2B). This result was supported when migration distance was replaced in the model with breeding latitude; species from higher latitudes had smaller erythrocytes (Table 1; Fig. 2C). The phylogenetic signal for these analyses was only moderate (Table 1).

**Table 1:** Phylogenetic mixed model analysis outputs of erythrocyte area, in relation to body mass, altitude, diving behavior, and either migration distance (km) or latitude

	Variable	Estimate	SE	*t*	*P*
Erythrocyte area	Body mass	0.12	0.01	11.00	<0.001
N = 631 species	Diving	0.07	0.03	2.41	0.016
λ = 0.62, R^2^ = 0.174	Altitude	−0.00	−0.00	−0.87	0.386
	Log migration distance	−0.003	0.002	−2.31	0.021
Erythrocyte area	Body mass	0.12	0.01	12.15	<0.001
N = 631 species	Diving	0.09	0.03	3.21	0.001
λ = 0.50, R^2^ = 0.220	Altitude	−0.00	−0.00	−0.45	0.652
	Latitude	−0.002	0.000	−5.51	<0.001
Elongation ratio	Diving	−0.03	0.03	−0.76	0.447
N = 597 species	Altitude	0.000	0.000	2.77	0.005
λ = 0.02, R^2^ = 0.03	Log migration distance	0.000	0.000	3.38	<0.001
Elongation ratio	Diving	−0.05	0.03	−1.65	0.098
N = 596 species	Altitude	0.000	0.000	2.26	0.024
λ = 0.01, R^2^ = 0.04	Latitude	0.001	0.000	4.56	<0.001

**Fig. 3:**
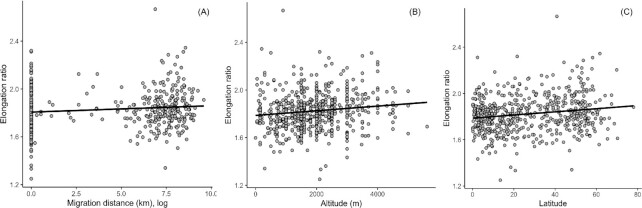
Effects of (**A**) total migration distance (log km), (**B**) altitude (m), and (**C**) absolute latitude (degrees) on erythrocyte elongation ratio.

Diving had no significant effect on elongation ratio of erythrocytes but species from high altitude habitats, and those that had long migration distances, were more elongated (Table 1; Fig. 3). The species from higher latitudes had more elongated erythrocytes and altitude remained a significant covariate (Table 1; Fig. 3). There were very low phylogenetic signals for these analyses (Table 1).

### Mating behavior and sexual selection

**Fig. 4:**
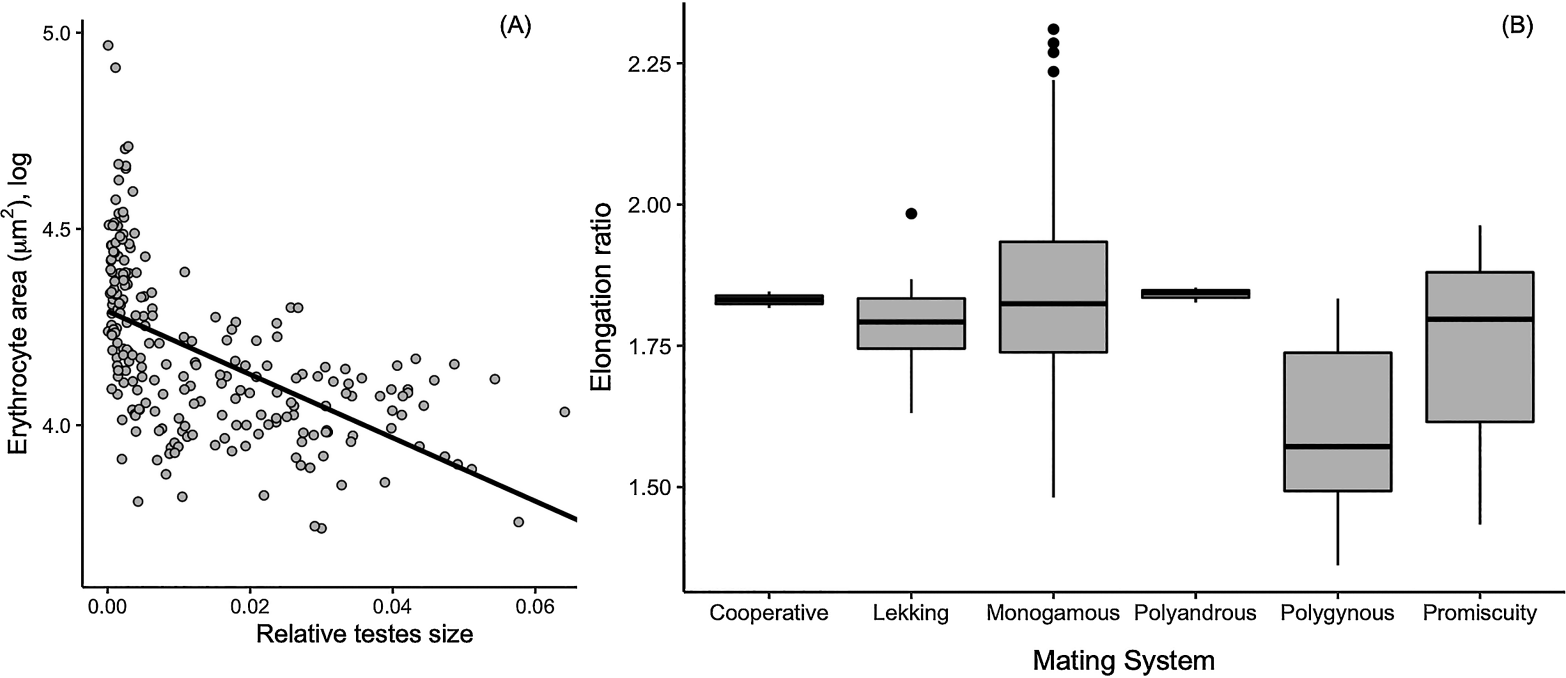
Effects of (**A**) relative testes size (testes mass/body mass) on erythrocyte area (μm²), and (**B**) mating system on erythrocyte elongation ratio.

We found no effect of mating system on erythrocyte size (Table 2), but species with larger relative testes sizes had smaller erythrocytes (Table 2; Fig. 4A). Elongation ratio was unrelated to relative testes size (Table 2), but polygynous species had smaller elongation ratios, i.e., rounder erythrocytes in comparison to polyandrous and cooperative breeding species (Table 2; Fig. 4B). Lambda values were relatively high for the erythrocyte size model indicating a good phylogenetic signal, but low for elongation ratio (Table 2).

**Table 2:** Phylogenetic mixed model analysis outputs for erythrocyte area and elongation ratio in relation to relative testes size and mating system

	Variable	Estimate	SE	*t*	*P*
Erythrocyte area	Relative testes size	−2.77	0.90	−3.09	0.002
N = 224	Mating system: lek	−0.05	0.10	−0.49	0.625
λ = 0.79, R^2^ = 0.03	Mating system: monogamous	−0.08	0.08	−0.99	0.322
	Mating system: polyandrous	−0.02	0.12	−0.14	0.893
	Mating system: polygynous	−0.12	0.10	−1.15	0.248
	Mating system: promiscuous	0.03	0.12	0.26	0.798
Elongation ratio	Relative testes size	1.01	0.69	1.46	0.144
N = 224	Mating system: lek	−0.04	0.10	−0.36	0.720
λ = 0.00, R^2^ = 0.05	Mating system: monogamous	0.01	0.09	0.06	0.953
	Mating system: polyandrous	−0.00	0.13	−0.02	0.987
	Mating system: polygynous	−0.23	0.11	−2.10	0.037
	Mating system: promiscuous	−0.11	0.13	−0.88	0.380

## Discussion

### Altitude and diving erythrocyte size in birds

Species classed as having a diving life style had significantly larger erythrocytes than non-diving species. Diving behavior is energetically costly (Butler 1991), but unlike exercise in air, birds rely on stored oxygen, which is primarily stored in the blood and muscles for long dives and the respiratory system for short dives (Kooyman and Ponganis 1998). Diving animals, including birds, have greater blood Hb levels (Minias 2020), which serve to store bound-oxygen. There is also increased hemoglobin-oxygen affinity and Bohr effect in penguins, which have the longest dives (Signore et al. 2021). Larger erythrocytes contribute significantly to binding more oxygen and therefore are critical for oxygen storage capacity. Evidence in diving mammals (Wickham et al. 1989; Davis 2014) and squamates (Penman et al., submitted for publication), show that diving species have larger erythrocytes than non-diving species, supporting the general effect of larger erythrocytes as an adaptation to diving.

Vertebrates that live at high altitude (above 2000 m) are subjected to hypoxic conditions that challenge aerobic metabolism. Birds are more tolerant of hypoxia than other terrestrial vertebrates (Lasiewski and Calder 1971; Faraci 1991). Even so, a bird's blood oxygen-carrying capacity show both plastic adaptations as well as evolutionary adaptations to high altitude life (Lague et al. 2016; Dawson et al. 2020; Minias 2020). Previous work has shown that erythrocyte size is smaller at higher altitude in squamates (Penman et al., submitted for publication), but we found no such relationship in birds. Instead, high altitude was associated with more elongated erythrocytes supporting evidence from within species that showed elongated erythrocytes were reported in alpine accentors *Prunella collaris* breeding at high altitudes (Janiga et al. 2017; Haas and Janiga 2020). High altitude squamates also have more elongated erythrocytes (Penman et al., submitted for publication). Ellipsoids maintain a larger surface area, which facilitates more efficient gas exchange than round cells (Hartman and Lessler 1963), and more elongated cells maintain surface area without increasing in breadth, which could hamper flow through capillary beds. Elliptical red blood cells may be characteristic of other high altitude adapted and probably facilitate effective oxygen transport at higher altitudes.

### Life history and mating behavior on erythrocyte size

We found that engaging in energetic activities such as reproductive competition and migration had significant effects on erythrocyte size and elongation ratio. First, we found that birds moving long distances during migration (and also breeding at higher latitudes) had smaller and longer erythrocytes. Smaller erythrocytes have larger surface area to volume ratios and shorter diffusion distances allowing more rapid oxygenation and deoxygenation of hemoglobin as erythrocyte volume decreases (Holland and Forster 1966; Jones 1979). The ability to deliver oxygen during exercise is a critical component of aerobic capacity (Brownscombe et al. 2017). This fits with a range of other physiological adaptations found in migrating birds, including larger effective areas for gas-exchange area and shorter diffusion distance, larger hearts, and greater capillary densities in the flight muscles (Lundgren and Kiessling 1988; McWilliams et al. 2004; Butler 2016). Smaller and longer erythrocytes will also facilitate transport through the capillary system of muscles (Noguchi and Gompper 2005), allowing more effective sustained aerobic activity necessary for flight.

We found a number of species with larger relative testes sizes had smaller erythrocytes, but that there were no clear patterns in mating system. Relative testes size is often used as a proxy of male–male postcopulatory competition, because of its relationship with both social and genetic mating systems (Pitcher et al. 2005; Soulsbury 2010). In addition, male mating strategies include many aerobically demanding activities (Soulsbury 2019), and there is some evidence that this may impact aerobic traits (e.g., differences in sexually dimorphic primates: Lindenfors et al. 2010). Experimentally, treating individuals with testosterone leads to higher HB levels, and smaller erythrocyte volumes (mean corpuscular volume) (Puerta et al. 1995), again suggesting a critical link between male–male competition and oxygen-carrying capacity. Shorter erythrocytes in relation to width, i.e., rounder erythrocytes, were found in species with greater mating competition (e.g., polygynous species). The reason for this is not clear, as more elliptical cells are more effective for oxygen exchange. Instead, rounder cells may reflect an increased population of young erythrocytes in circulation, as higher androgen levels increase erythropoiesis rates (Puerta et al. 1995; Nikinmaa 2020). In any case, we suggest that oxygen-carrying capacity is an important trait under sexual selection due to its strong underpinning of male performance.

## Conclusions

This study explored the shape and size of avian erythrocytes and explored how energetic lifestyle may select on different sizes and shapes. In general, we found that energetically costly activities led to smaller erythrocytes, which facilitate oxygen transport and exchange. The exception was diving birds, which had larger erythrocyte as they use bound-oxygen as an oxygen store during diving. This study emphasizes the important role of avian life history on driving the size and shape of the avian erythrocyte.

## Data accessibility

The dataset is located at FigShare (doi:10.6084/m9.figshare.16545852).
